# Gut microbiota, serum metabolites, and lipids related to blood glucose control and type 1 diabetes

**DOI:** 10.1111/1753-0407.70021

**Published:** 2024-10-27

**Authors:** Zhaohe Gu, Lanxin Pan, Huiling Tan, Xulin Wang, Jing Wang, Xueying Zheng, Jianping Weng, Sihui Luo, Tong Yue, Yu Ding

**Affiliations:** ^1^ Department of Endocrinology, The First Affiliated Hospital of USTC, Division of Life Sciences and Medicine, Clinical Research Hospital of Chinese Academy of Sciences (Hefei) University of Science and Technology of China Hefei China

**Keywords:** glycemic control, gut microbiome, lipidomics, metabolomics, type 1 diabetes mellitus

## Abstract

**Background:**

The composition and function of gut microbiota, lipids, and metabolites in patients with type 1 diabetes (T1D) or its association with glycemic control remains unknown. We aimed to use multi‐omics sequencing technology and machine learning (ML) approaches to investigate potential function and relationships among the gut microbiota, lipids, and metabolites in T1D patients at varied glycemic levels.

**Methods:**

We conducted a multi‐omics analysis of the gut microbiome from fecal samples, metabolites, and lipids obtained from serum samples, collected from a cohort of 72 T1D patients. The patients were divided into two groups based on their hemoglobin A1c (HbA1c) levels. 16S rRNA sequencing, and metabolomics methods were applied to analyze changes in composition and function of gut microbiota, metabolites, and lipids.

**Results:**

The linear discriminant analysis, Shapley additive explanations (SHAP) algorithm, and ML algorithms revealed the enrichment of *Bacteroides_nordii, Bacteroides_cellulosilyticus* in the glycemic control (GC) group, while *Bacteroides_coprocola* and *Sutterella_wadsworthensis* were enriched in the poor glycemic control (PGC) group. Several metabolic enrichment sets like fatty acid biosynthesis and glycerol phosphate shuttle metabolism were different between two groups. *Bacteroides_nordii* exhibited a negative association with D‐fructose, a component involved in the starch and sucrose metabolism pathway, as well as with monoglycerides (16:0) involved in the glycerolipid metabolism pathway.

**Conclusions:**

We identified distinct characteristics of gut microbiota, metabolites, and lipids in T1D patients exhibiting different levels of glycemic control. Through comprehensive analysis, microbiota (*Bacteroides_nordii*, *Bacteroides_coprocola*), metabolites (D‐fructose), and lipids (Monoglycerides) may serve as potential mediators that communicated the interaction between the gut, circulatory systems, and glucose fluctuations in T1D patients.

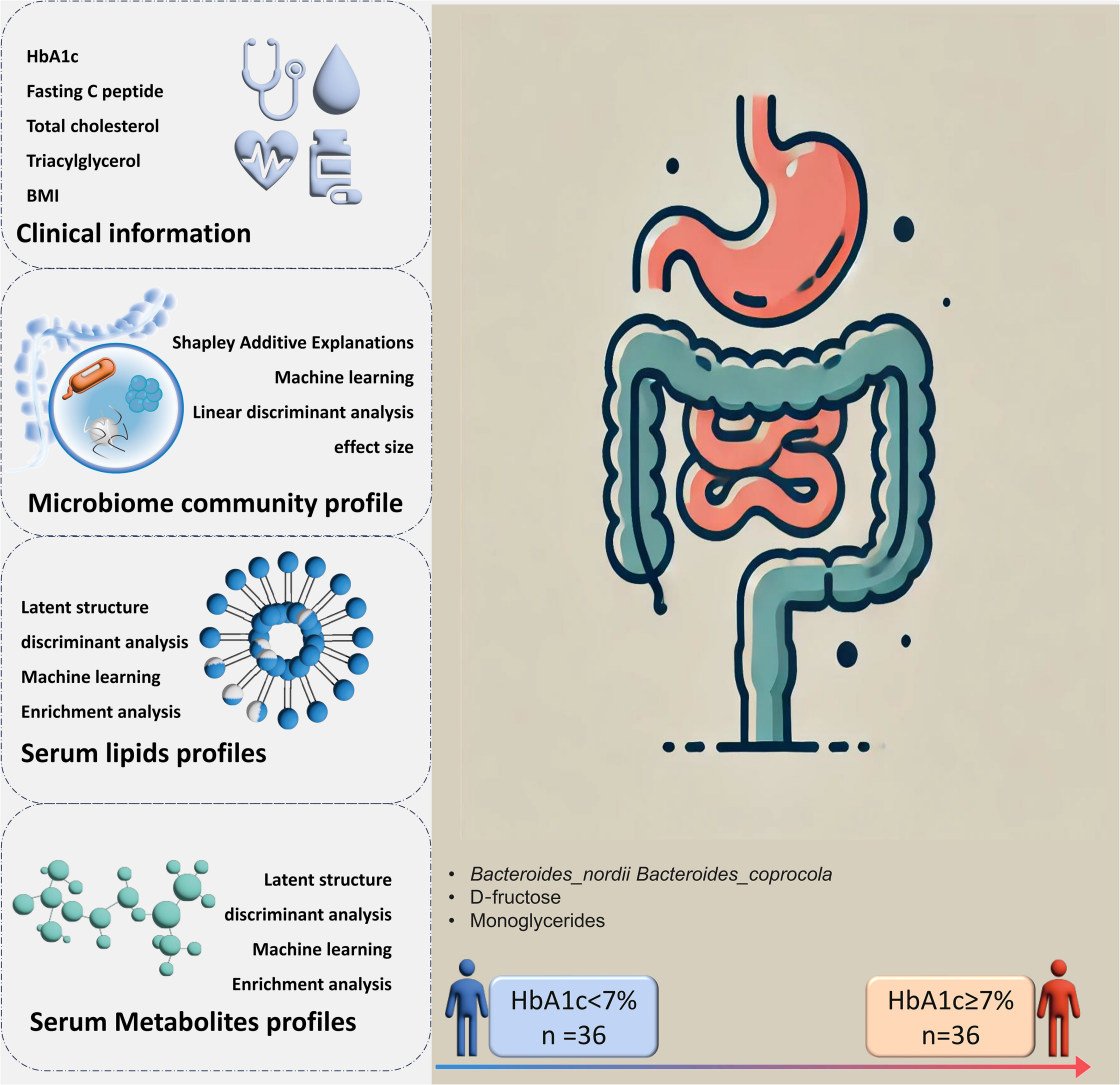

## Graphical abstract

- Our integrated multi‐omics analyses revealed the potential glycemic control–microbiota–metabolite/lipid–T1D relationship.
- We identified several bacterial genera (e.g., *Bacteroides_nordii*, *Bacteroides_cellulosilyticus*) positively associated with the well glycemic control linked to T1D.
- Through comprehensive analysis, microbiota (*Bacteroides_nordii*, *Bacteroides_coprocola*), metabolites (D‐fructose), and lipids (Monoglycerides) may serve as potential mediators that communicated the interaction between the gut, circulatory systems, and glucose fluctuations in T1D patients.

## 1 INTRODUCTION

Type 1 diabetes (T1D) is an autoimmune disease characterized by the immune‐mediated destruction of pancreatic beta cells,^1^ resulting in insulin deficiency and uncontrolled glucose levels.^2^ Achieving optimal glucose control is pivotal in managing T1D and mitigating the risk of associated complications, which encompass a wide range of cardiovascular, renal, and neurological disorders.^3^, ^4^, ^5^ The Diabetes Control and Complications Trial (DCCT) found that in T1D patients,^6^ intensive glycemic control (mean A1C about 7%) compared to standard control (mean A1C about 9%) significantly reduced rates of microvascular complications development and progression by 50%–76%.

The gut microbiota influences host metabolism and is involved in the early metabolic disturbances during the progression of T1D.^7^ There are differences in the gut microbiome between T1D patients and healthy individuals.^8^ Importantly, mounting evidence suggests a link between dysregulated gut microbiota and disrupted blood glucose control.^9^, ^10^ In patients with type 2 diabetes, those with higher abundance of *Bacteroides* species in the gut microbiota exhibit better blood glucose control.^11^ Furthermore, studies have found differences in various serum metabolites and lipid molecules between T1D patients and healthy individuals.^12^ Short‐chain fatty acids (SCFAs), metabolites of the gut microbiota, can reduce fasting blood glucose and lipid levels in T1D mice and patients,^13^, ^14^ while some metabolites can lower blood glucose levels in type 2 diabetes mice.^15^ However, there is currently no research observing the correlation between different gut microbiota profiles and different levels of blood glucose control in T1D patients with various metabolites/lipids. Therefore, the interactions among blood glucose regulation, gut microbiota, serum lipids, and metabolites in T1D patients' bodies, influencing blood glucose management, are largely unexplored and not well understood.

Prior research has primarily noted the connection between gut microbiota and hemoglobin A1c (HbA1c) levels among T1D patients.^16^, ^17^, ^18^ A study classified individuals into two groups based on HbA1c levels (<7.5% and ≥7.5%) and examined the association between continuous subcutaneous insulin infusion (CSII) therapy, dietary habits, and HbA1c levels in T1DM participants.^19^ Another study, stratifying T1D patients receiving guided insulin pump therapy into two groups based on HbA1c levels: <7.0% and ≥7.0%, observed blood glucose control and gut microbiota profiles.^20^ We are categorizing T1D patients based on the recommended glycemic control target (<7.0% HbA1c) outlined in guidelines, aiming to investigate multi‐omics differences (including gut microbiota, metabolites, and lipids) for the first time.^3^, ^21^

In this study, we employ a multi‐omics strategy to elucidate the distinct features associated with glycemic control in individuals with T1D. Leveraging gut microbiome and metabolomic and lipidomic analyses, we strive to identify key gut microbiome, metabolites, and lipids linked to glucose regulation, facilitating the exploration of multi‐omics variances between these groups and providing critical insights into potential pathways crucial for glycemic regulation.

## 2 METHODS

### 2.1 Participant recruitment and sample collection

In this cross‐sectional study, we recruited 101 patients from the Department of Endocrinology at the Third Affiliated Hospital of Sun Yat‐sen University from 2019 to 2020.^22^ The diagnosis of T1D was made based on the criteria outlined by the American Diabetes Association.^23^

Exclusion criteria covered chronic gastrointestinal disorders, recent use of antibiotics, probiotics, or corticosteroids within 3 months post‐enrollment, both chronic and acute inflammatory and infectious conditions, as well as pregnancy and breastfeeding. We defined glycemic control (GC) using the recommended glucose control targets by IPSAD and ADA as having HbA1c <7%. Glycemic control not meeting the criteria is defined as PGC (poor glycemic control, HbA1c ≥7%).^3^, ^21^

In result, 36 T1D patients with well glycemic control (GC group) were recruited to match 36 T1D patients with poor glycemic control (PGC group). Clinical assessment with blood draws and fecal sample collection from each participant were conducted at in‐person clinic visit.

### 2.2 Gut microbiome 16S rRNA gene sequence analysis

Fecal samples were processed for DNA extraction using the MagPure Stool DNA KF kit B (Magen, China). PCR amplification of the V3–V4 regions of the bacterial 16S rRNA gene was conducted using primers 806R and 341F. AmpureXP beads were employed for the purification of amplicons, followed by paired‐end sequencing performed on the Illumina platform. Raw sequencing data were processed using the Quantitative Insights Into Microbial Ecology (QIIME2).^24^ Sequence assembly was performed with FLASH (Fast Length Adjustment of Short reads, v1.2.11),^25^ where paired‐end reads were merged based on overlapping regions to generate single sequences representing hypervariable region tags. Tags were clustered into operational tax units (OTUs) by using USEARCH (v7.0.1090) with a 97% similarity cutoff.^26^ Chimeric sequences were removed using UCHIME (v4.2.40) prior to further analysis.^27^ Additionally, high‐quality sequences were processed to generate amplicon sequence variants (ASVs) using the DADA2 (Divisive Amplicon Denoising Algorithm) method within QIIME2.^28^ The representative sequence of each OTU was then annotated with taxonomic information based on the SILVA database (v138, released December 16, 2019),^29^ using the Mothur algorithm (v1.44.2).^30^

We utilized various alpha diversity indices like the Chao1 index and the Shannon diversity index to evaluate differences in gut microbial community abundance between the GC and PGC groups. Beta diversity comparisons were conducted between the two groups to assess global differences in microbiota composition and structure, based on ASV abundance. These analyses were performed via the MicrobiomeAnalyst website.^31^, ^32^ Additionally, linear discriminant analysis effect size (LEfSe)^33^ was also employed on the MicrobiomeAnalyst to identify significantly different taxa between the GC and PGC groups. The Shapley additive explanations (SHAP) algorithm values of the data were calculated using the TreeExplaine, implemented in Python 3.8.^34^

### 2.3 Serum metabolomics and lipidomics analysis

The metabolite and lipid extraction process closely followed established protocols.^35^, ^36^ Samples (100 μL) were extracted in a 2:1 (v/v) mixture with 300 μL of pre‐cooled methanol and acetonitrile, along with a combination of internal standards (IS1 and IS2) for sample preparation quality control. After vortexing for 1 min, the samples were incubated at −20°C for 2 h, followed by centrifugation at 4000 rpm for 20 min. The resulting supernatant was transferred for vacuum and freeze‐drying. Dried metabolites were reconstituted in 150 μL of 50% methanol and centrifuged at room temperature for 30 min. The supernatant was transferred into autosampler vials for LC–MS analysis. A quality control sample, pooling equal volumes of individual samples, was prepared to assess the overall repeatability of the LC–MS analysis.

Metabolomics and lipidomics analyses were conducted using the MetaboAnalyst website.^37^ Orthogonal projections with orthogonal projections to latent structures discriminant analysis (OPLS‐DA) were utilized to evaluate the overall metabolic distribution and identify differential metabolites between the GC and PGC groups; the same is true of lipidomics analysis. Differential serum metabolites and lipids were determined based on fold changes (>1.2) and *p*‐values (<0.05). The signaling pathways and biochemical metabolic pathways associated with these differential metabolites were annotated using MetaboAnalyst.^38^ We applied four machine learning algorithms (logistic regression [LR], support vector machine [SVM], Gaussian naive Bayes [GNB], and random forest [RF]) in Python to identify the differing metabolites or lipids between GC and PGC.

### 2.4 Correlational analysis of microbiome and metabolome/lipidome

We employed Spearman correlation analysis to investigate the intricate relationship between the microbiome and metabolome/lipidome through connectivity network analysis. Differential metabolites/lipids and microbiota identified earlier were selected to compute correlation coefficients and statistical significance using the R package psych 2.1.9.

### 2.5 Statistical analysis

Statistical analyses were performed using R version 4.2.3 (http://www.r-project.org/) and Python 3.8 (https://www.python.org/). We utilized the R package Comparison Group 4.5.1 to analyze clinical characteristics. We utilized the TreeExplainer implemented in Python 3.8 to calculate the SHAP values of the data. We first used the Shapiro–Wilk test to check the normal distribution of continuous variables. Then, we compared continuous variables between groups using *T*‐tests or analysis of variance (ANOVA). Specific statistical methods and websites were utilized for microbiome and metabolome data analyses, as mentioned previously. A significance level of *p* values <0.05 was considered.

## 3 RESULTS

### 3.1 Clinical characteristics

In this study, 72 patients with T1D were enrolled. Recruitment details can be found in the methods section. These patients were divided into two groups based on their glycemic control: Well glycemic control group (GC, 33.3% male) and poor glycemic control group (PGC, 41.6% male).

The well glycemic control is defined as HbA1c <7.0%. No significant differences were found between the groups in terms of gender, body mass index, total cholesterol, total calories, and LDL‐cholesterol. As expected, GC had significantly lower levels of HbA1c. Additional clinical characteristics are detailed in Table 1 for further reference and analysis.

**TABLE 1:** Clinical characteristics of the participants.

	GC	PGC	*p* overall
	*N* = 36	*N* = 36	
Age (year)	31.9 (9.80)	31.1 (10.8)	0.733
Male	12 (33.3%)	15 (41.6%)	0.472
BMI (kg/m^2^)	20.66 (1.84)	21.25 (2.70)	0.285
TC (mmol/L)	4.75 (0.82)	4.80 (0.84)	0.779
TG (mmol/L)	0.79 (0.31)	0.79 (0.38)	0.922
HDLC (mmol/L)	1.57 (0.31)	1.56 (0.29)	0.897
LDLC (mmol/L)	2.69 (0.70)	2.89 (0.67)	0.226
HbA1c (%)	6.12 (0.50)	8.09 (1.52)	<0.001
Fasting C peptide (nmol/L)	0.05 (0.06)	0.04 (0.05)	0.456
Islet autoantibodies (+)	21 (58.3%)	25 (69.4%)	0.462
GADA (+)	14 (38.9%)	22 (61.1%)	0.099
ZnT8A (+)	6 (16.7%)	6 (16.7%)	1.000
IA2A (+)	10 (27.8%)	10 (27.8%)	1.000
Age of onset (year)	19.97 (8.80)	18.39 (10.6)	0.492
Diabetes duration (year)	11.91 (5.74)	12.62 (6.61)	0.630
Alcohol (yes)	8 (22.2%)	15 (41.6%)	0.079
Smoking (yes)	2 (5.56%)	6 (16.67%)	0.260

*Note*: Data are presented as number (%) and median (interquartile range). The normal reference ranges for the clinical parameters mentioned are as follows: TC (3.6–5.2 mmol/L); TG (0.6–2.3 mmol/L); HDLC (≥1.0 mmol/L); LDLC (<3.4 mmol/L).

Abbreviations: BMI, body mass index; GADA, anti‐glutamic acid decarboxylase antibodies; HbA1c, hemoglobin A1c; HDLC, high‐density lipoprotein cholesterol; IA2A, insulinoma associated‐2 autoantibodies; LDLC, low‐density lipoprotein cholesterol; TC, total cholesterol; TG, triacylglycerol; ZnT8A, zinc transporter 8 autoantibodies.

### 3.2 Microbiome community profiling of GC and PGC

**FIGURE 1:**
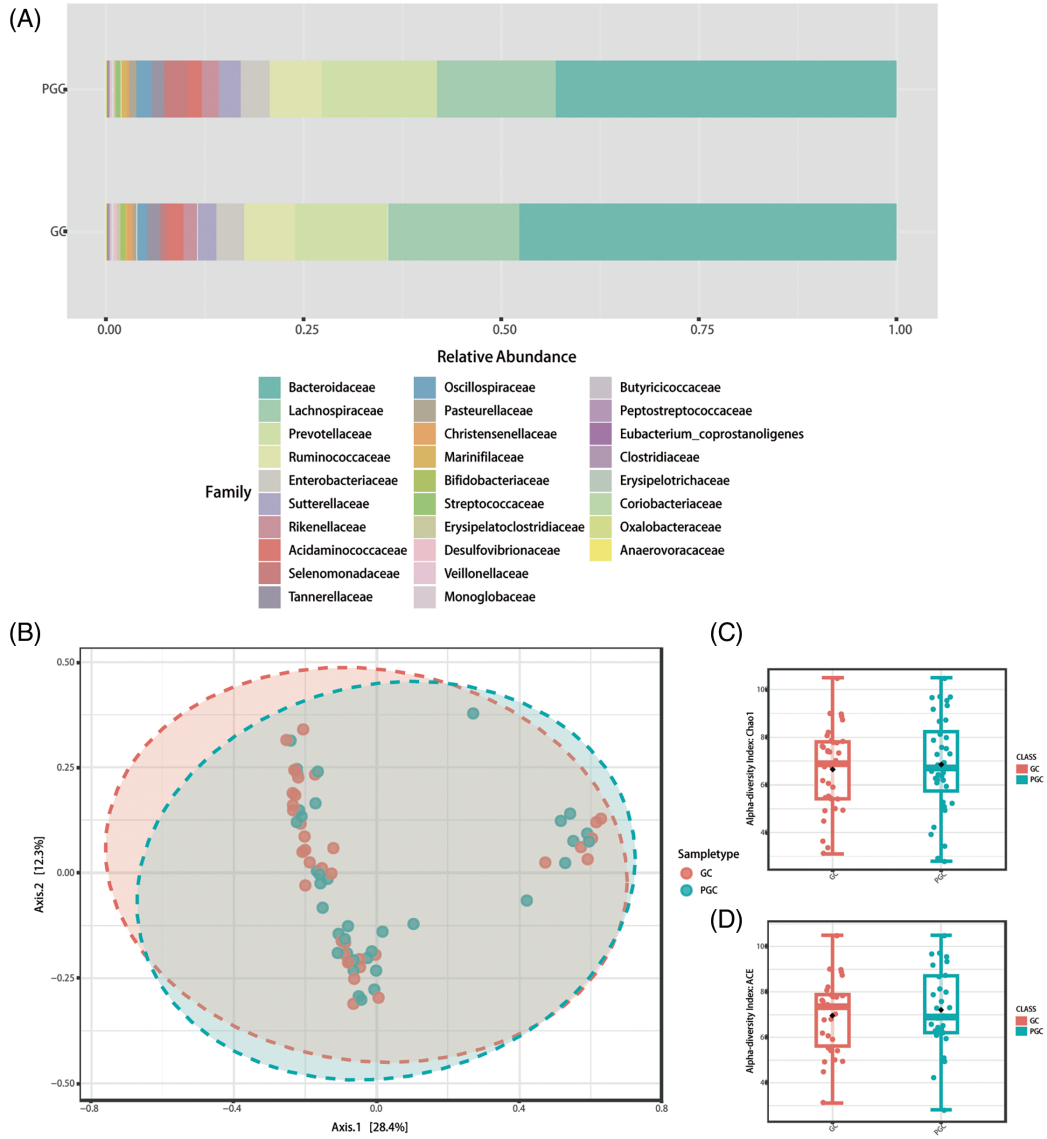
Results of diversity and taxonomy. (A) At the family level, the stacked bar plots. (B) Result of β‐diversity visualized using principal coordinate analysis based on Bray–Curtis Index (permutational MANOVA test, *F* = 0.92729, *R* ^2^ = 0.013074, *p* = 0.4520). (C, D) The plots of α‐diversity. (C) The Chao1 diversity boxplots (*T*‐test, *p* = 0.5903). (D) The ACE index boxplots (*T*‐test, *p* = 0.7115).

Figure 1A illustrates the summary of relative abundances at the family levels. Other taxonomic level results, including phylum, class, order, genus, and species, are presented in Figure S1A–E. At the phylum level, both GC and PGC groups are primarily dominated by two phyla: *Bacteroidetes* and *Firmicutes*. However, *Proteobacteria* appears more abundant in the GC group, whereas *Actinobacteria* shows a slightly higher relative abundance in the PGC group.

Within the class level, *Bacteroidia* and *Clostridia* are predominant in both groups. Notably, *Gammaproteobacteria* has a higher relative abundance in the GC group compared to the PGC group. *Negativicutes* and *Bacilli* also show variation between the groups, with *Bacilli* being slightly more represented in the PGC group. At the order level, *Bacteroidales* and *Clostridiales* are the most abundant orders in both groups. *Enterobacteriales* (which belongs to *Gammaproteobacteria*) is more prevalent in the GC group, while *Lactobacillales* (from *Bacilli*) is more dominant in the PGC group. Genus‐level analysis shows that *Prevotella* and *Bacteroides* are significantly abundant across both groups. *Escherichia* (from *Enterobacteriaceae*) has a notably higher presence in the GC group, while *Lactobacillus* is more represented in the PGC group. At the species level, certain species within the genera *Bacteroides* and *Prevotella* are highly abundant in both groups.

In α‐diversity analysis, both the ACE index (*T*‐test, *p* = 0.7115) and the Chao1 index (*T*‐test, *p* = 0.5903) exhibited a slight but nonsignificant increase between PGC and GC groups (Figure 1C,D). Figure S1F–H present results of the Shannon index (*T*‐test, *p* = 0.7873), Simpson index (*T*‐test, *p* = 0.7294), and Fisher index (*T*‐test, *p* = 0.4424). Although graphical differences are observable, none reached statistical significance between the two groups. β‐diversity plots can be seen in Figure 1B, indicating no significant difference between groups (permutational multivariate analysis of variance test, *F* = 0.92729, *R* ^2^ = 0.013074, *p* = 0.4520).

**FIGURE 2:**
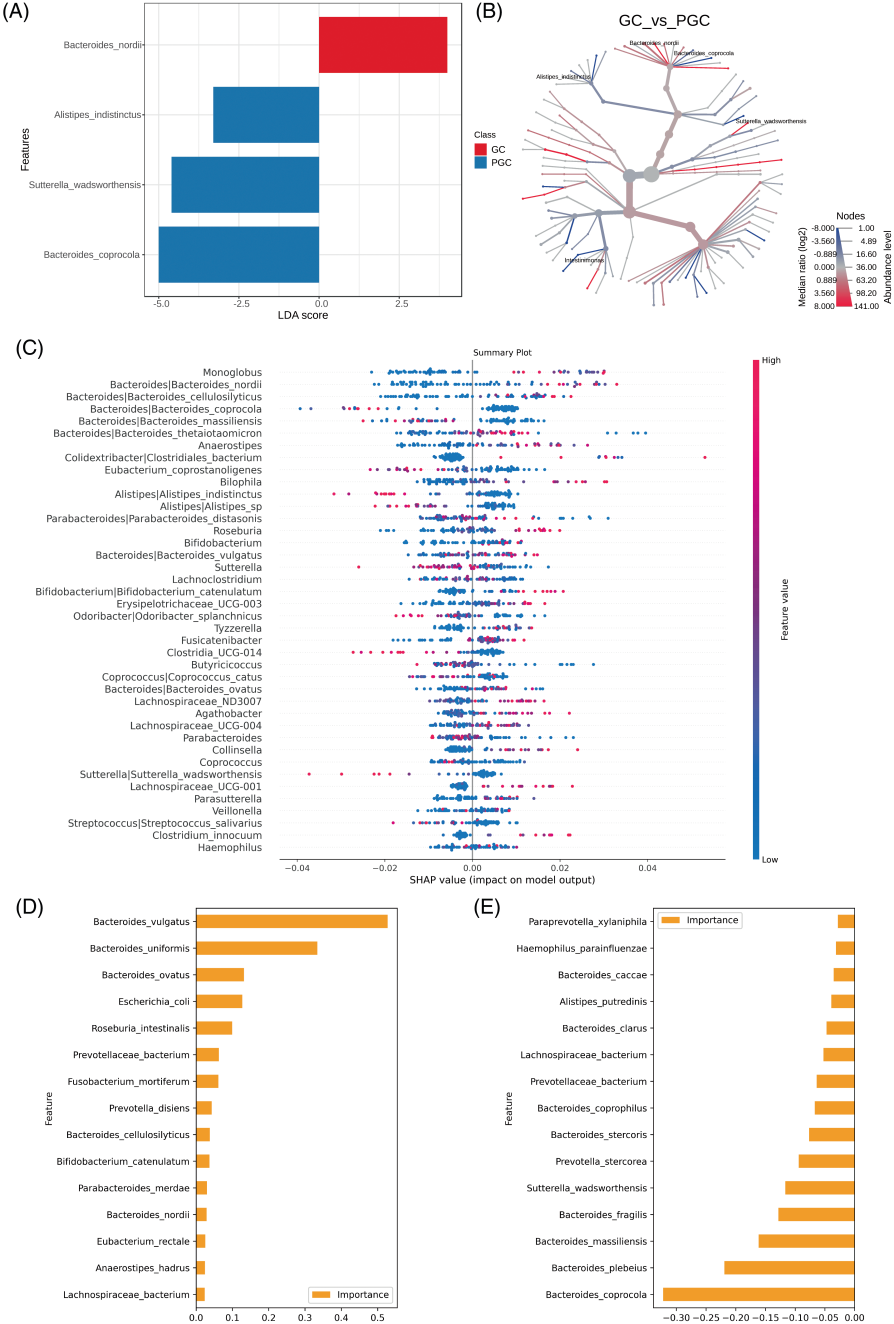
Results of different taxa (screened by *p* value < 0.05) between two groups by LEfSe analysis. (A) Histogram of LDA value distribution. (B) Cladogram plots depicting the microbial taxa that differed significantly. (C) SHAP summary plot depicting the top 40 microbial features. (D) The top 15 microbial features predicted by ML that contribute to the well glycemic control. (E) The top 15 microbial features predicted by ML that contribute to the poor glycemic control.

We subsequently analyzed the distinct microbiota composition between the GC group and PGC group employing LEfSe. Discriminative features were identified with a linear discriminant analysis (LDA) score threshold of 1.2. A total of four bacterial species were significantly enriched in two groups. The histogram of LDA value distribution and the cladogram of different taxa are demonstrated in Figure 2A,B. *Bacteroides_nordii* (Wilcox *p* value = 0.0092) and *Bacteroides_coprocola* (Wilcox *p* value = 0.0011) exhibit the most notable disparity between the GC and PGC groups, respectively. The identification of specific bacterial species that are enriched in either the GC or PGC groups provides insights into potential microbial markers for glycemic control. *Bacteroides_nordii*, which is more abundant in the GC group, might play a beneficial role in glucose metabolism, while *Bacteroides_coprocola*'s higher abundance in the PGC group could be associated with poorer glycemic outcomes.

We applied the TreeExplainer framework by Lundberg et al. to compute and visualize SHAP values. Local explanations for three randomly selected subjects are depicted in Figure S2(A–C).^34^ To understand the influence direction, we visualized the SHAP values of the top 40 features using beeswarm plots, referred to as the summary plot (Figure 2C). The *x*‐axis position represents the bacterial species' influence on the classifier predictions for each feature. Colors indicate original feature values (relative abundance), with blue indicating low and red indicating high abundance. Notably, *Bacteroides_nordii* and *Bacteroides_cellulosilyticus* exhibit a trend where high relative abundance (red) aligns with positive *x*‐axis values, suggesting a higher likelihood of GC. Conversely, lower abundances (blue) are associated with a lower GC probability. Conversely, *Bacteroides_coprocola* and *Sutterella_wadsworthensis* show an inverse pattern, indicating that higher abundance corresponds to a lower GC probability and vice versa. Additionally, we employed LR, GNB, SVM, and RF, suitable for limited sample sizes, to identify differential microbes. Combining SHAP and machine learning (ML) algorithms enhances the screening of strict microbial biomarkers.

### 3.3 Circulating metabolite profiles of GC and PGC

**FIGURE 3:**
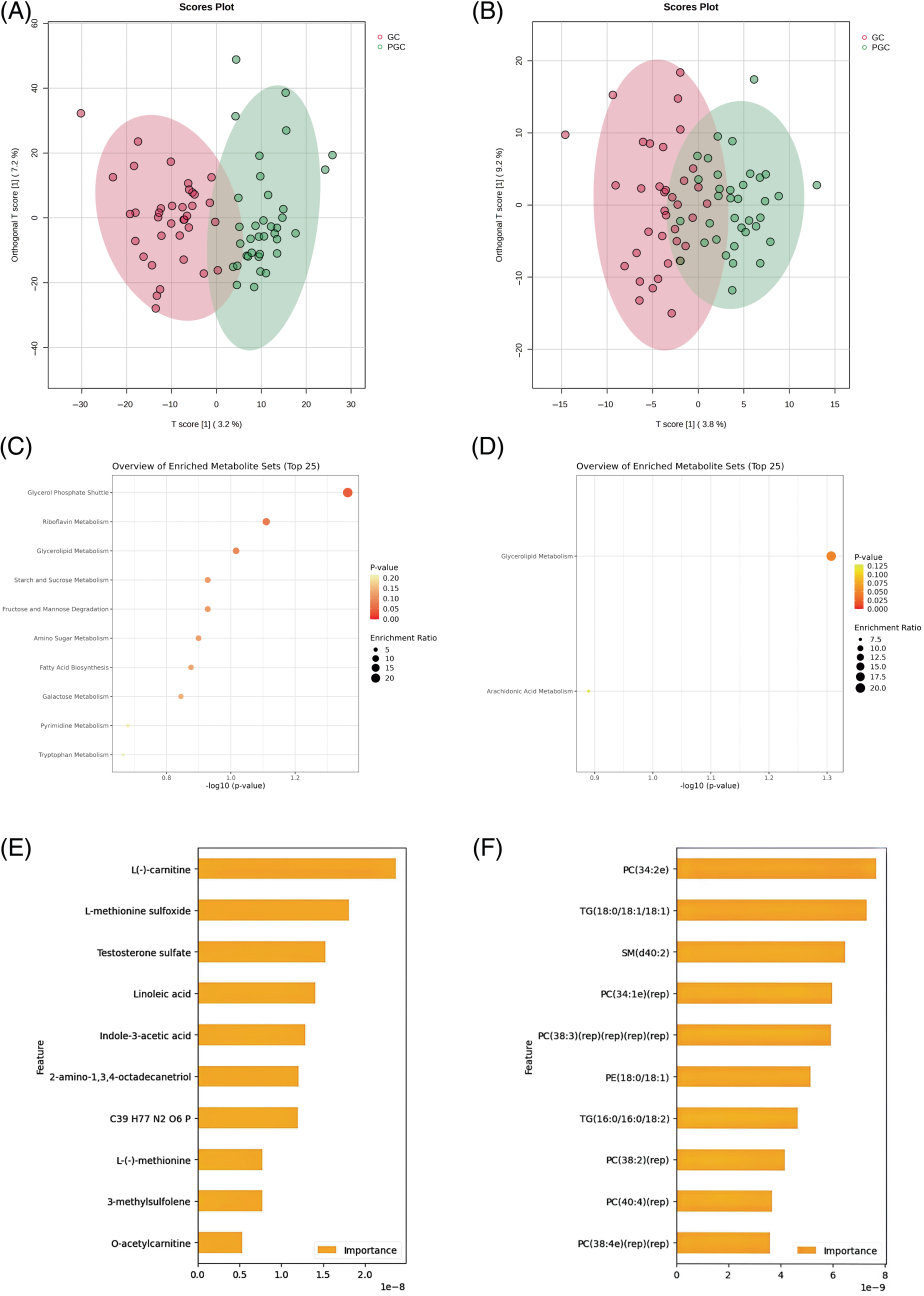
Results of metabolites profiles analysis between two groups. (A) OPLS‐DA analysis displaying a discriminative trend of metabolite composition and (B) lipid composition between two groups. (C) Differential metabolites (screened by combining *p* value [<0.05] and fold change [>1.2]) mainly involved in glycerol phosphate shuttle enrichment. (D) Differential lipids (screened by combining *p* value [<0.05] and fold change [>1.2]) mainly involved in glycerollipid metabolism enrichment. (E) The top 10 metabolic features predicted by ML that contribute to the well glycemic control. (F) The top 10 lipidomic features predicted by ML that contribute to the well glycemic control.

The two groups displayed markedly distinct compositions of serum lipids and metabolites, as discerned through OPLS‐DA. Additionally, we identified 441 differential metabolites and 36 lipids using criteria based on fold change (>1.2) and *p*‐value (<0.05). These findings are depicted in volcano plots presented in Figure S3A,B. We utilized ML algorithms (LR, GNB, SVM, and RF) to identify altered metabolic signatures in GC, depicted in Figure 3E,F. Subsequently, we combined the upregulated metabolites in GC, the top 100 candidate biomarkers identified by ML, and the increased differential metabolites identified in OPLS‐DA. Similarly for downregulated metabolites. In comparison to the PGC group, Α‐l‐fucopyranose and L‐rhamnose exhibited decreased levels in the GC group. The differences between the two groups for Α‐l‐fucopyranose and L‐rhamnose are illustrated in the violin plots presented in Figure S4A. We then conducted enrichment analysis of metabolites to further elucidate their biological significance. The enrichment results of differential serum metabolites are illustrated in Figure 3C, revealing the top three enriched pathways as follows: glycerol phosphate shuttle (*p* = 0.0218), riboflavin metabolism (*p* = 0.0399), and glycerolipid metabolism (*p* = 0.0499). Furthermore, pathways enriched with upregulated metabolites included fatty acid biosynthesis (*p* = 0.0687) and tryptophan metabolism (*p* = 0.1140), as depicted in Figure S4B. The enrichment analysis further supports the relevance of these metabolic pathways in distinguishing between the two groups, underscoring the importance of integrated metabolic and microbial analyses in understanding disease mechanisms.

### 3.4 Serum lipid profiles of GC and PGC

In this study, a comparative analysis of lipid profiles between the GC and PGC groups revealed significant differences. According to OPLS‐DA analysis, differential lipidomic profiles were observed between the GC and PGC groups (Figure 3B); the majority of differential lipids were phosphatidylcholines. Compared to the PGC group, the GC group exhibited upregulation of 28 lipids, while downregulation was noted in PC(14:0/20:4), MG(16:0), PC(34:5), LPC(19:3), PS(38:6p), PE(16:0p/20:4)(rep), PE(36:4p), and PE(16:0p/20:4)(rep)(rep). To further understand the lipidomic changes associated with glycemic control, machine learning algorithms were employed to further examine the altered lipidome in GC. The top 10 upregulated lipids included PC(34:2e), TG(18:0/18:1/18:1), SM(d40:2), PC(34:1e)(rep), PC(38:3)(rep)(rep)(rep)(rep), PE(18:0/18:1), TG(16:0/16:0/18:2), PC(38:2)(rep), PC(40:4)(rep), and PC(38:4e)(rep)(rep). Notably, PC(38:2)(rep) was also among the top 10 differential lipids based on fold change (Figure 3F).

Subsequent pathway enrichment analysis of the upregulated lipids revealed that these lipids were significantly associated with metabolic pathways such as glycerolipid metabolism (*p* = 0.0493) and arachidonic acid metabolism (*p* = 0.1290). The involvement of these pathways suggests that altered lipid metabolism could contribute to differences in glycemic control among T1D patients, particularly through the modulation of glycerolipid and arachidonic acid metabolic processes, as indicated by the specific lipids MG(0:0/16:0/0:0) and PC(34:4).

### 3.5 Correlation between serum metabolites/lipids and gut microbiota

**FIGURE 4:**
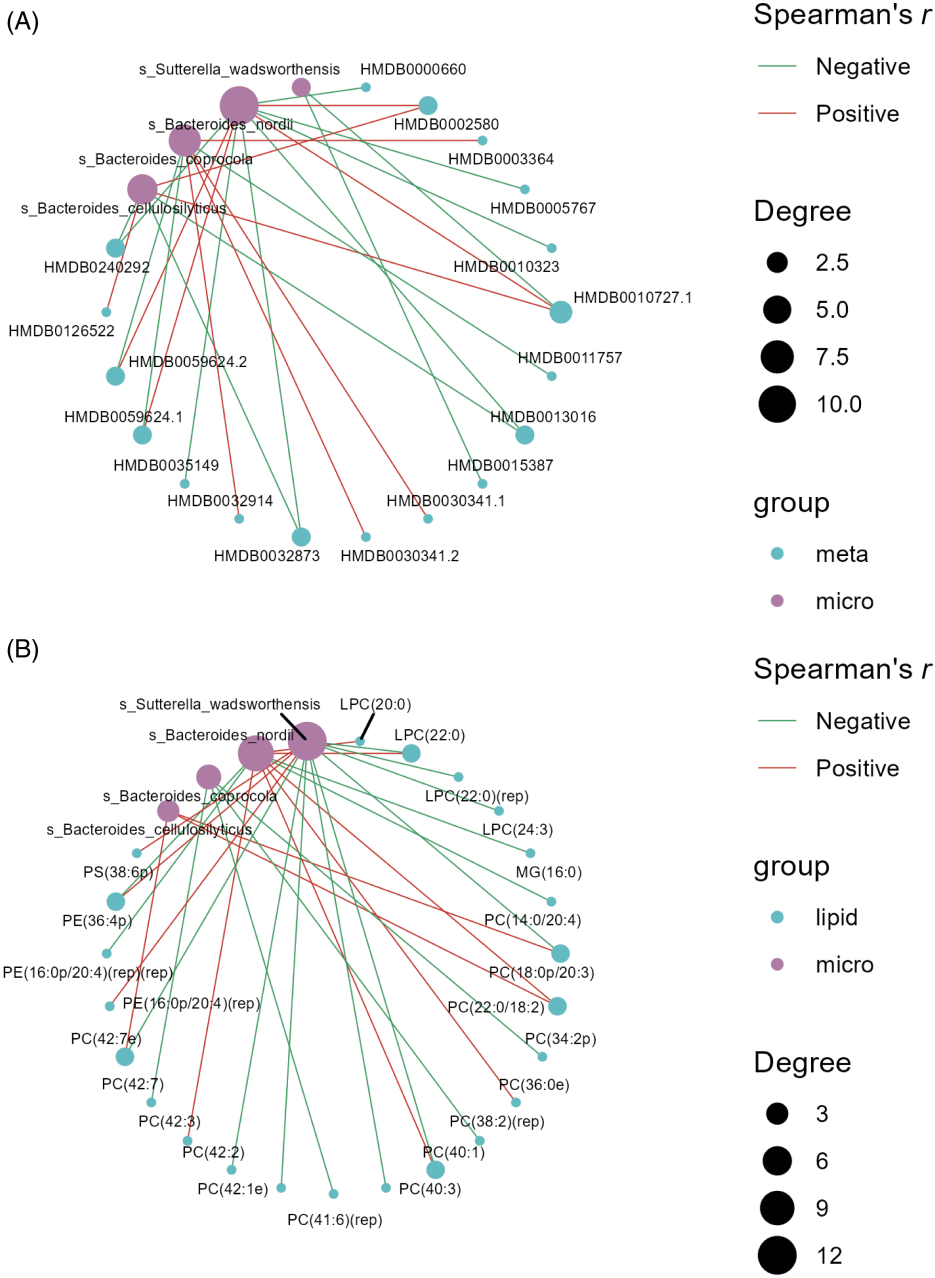
Results of combined analysis between two groups. (A) Results of combined analysis of microbiome and metabolome between two groups. (B) Results of combined analysis of microbiome and lipidome between two groups.

Analysis revealed positive associations between glycemic control, differential gut microbiota, and differential serum metabolites, notably linking *Bacteroides_nordii* and *Bacteroides_cellulosilyticus* with 3‐oxolauric acid (HMDB0010727), as depicted in Figure 4A. *Bacteroides_nordii* was positively associated with taurolithocholic acid 3‐sulfate (HMDB0002580) and negatively associated with D‐Fructose (HMDB0000660, hit by starch and sucrose metabolism), MG(16:0) (hit by glycerolipid metabolism) and PC(14:0;20:4). The compound benzoquinone (HMDB0003364) is associated with multiple enriched pathways, including glycerol phosphate shuttle, riboflavin metabolism, and glycerolipid metabolism. Additionally, it is positively correlated with *Bacteroides_coprocola*. In the GC group, the increased lipid PC(38:2)(rep) displayed a negative correlation with *Bacteroides_coprocola* observed in the PGC group.

## 4 DISCUSSION

In this study, we analyzed the gut microbial composition, examined metabolomic and lipidomic profiles of adults with T1D, and identified associations between these multi‐omics profiles and glycemic control status in patients with T1D.

Previous studies have demonstrated that environmental factors, including gut microbiota and metabolites, influence the pathogenesis of T1D and have linked the differential gut microbiota, serum lipids and metabolites observed between the GC group and PGC group to the development of T1D and other autoimmune diseases in humans. In our study, the overlapping microbes identified through ML and SHAP include *Bacteroides_nordii*, *Bacteroides_cellulosilyticus*, *Bacteroides_coprocola*, and *Sutterella_wadsworthensis*. We discovered that GC patients had a predominance of *Bacteroides_nordii* and *Bacteroides_cellulosilyticus*. PGC patients had a predominance of *Bacteroides_coprocola* and *Sutterella_wadsworthensis. Bacteroides_nordii* are found to be related to diabetes and the other human autoimmune diseases in previous studies inflammatory bowel diseases,^39^ allergic rhinitis.^40^ Yaowen Chen et al.^41^ found that *Bacteroides.coprocola* had a characteristic distribution of SNPs in the T2D patient group. This study found that the genes ranked first and sixth in *Bacteroides_coprocola* encode glycoside hydrolases. This seems to suggest an association of this fungus with glucose metabolism disruption.

*Bacteroides_cellulosilyticus* has been reported to be abundant T1D pediatric patients in Italy^42^; however, it is a multifunctional carbohydrate‐degrading microorganism. Its relative abundance is positively correlated with the overall abundance of microbial glycoside hydrolases in the infant gut.^43^ In our research, the observed enrichment of *Bacteroides_cellulosilyticus* in the GC group implies its potential role in influencing glycemic control and its positive association with 3‐oxolauric acid. This effect on glycemic regulation may be attributed to its ability to upregulate microbial glycoside hydrolases in the gut.^44^

*Bacteroides_nordii* is negatively associated with D‐fructose (HMDB0000660, hit by starch and sucrose metabolism). In the D‐fructose‐fed mouse model of non‐alcoholic fatty liver disease (NAFLD) induction, a decrease in the *Bacteroidetes* phylum was observed.^45^ This finding is consistent with the negative correlation observed between *Bacteroides_nordii* and D‐fructose in our study, indicating a potential association of *Bacteroides_nordii* with carbohydrate metabolism pathways.

In Tan et al.'s study, it was found that HbA1c levels were positively correlated with six bacterial taxa: *Ruminococcus torques*, *Lactobacillales*, *Streptococcaceae*, *Bacilli*, *Erysipelotrichales*, and *Erysipelatoclostridiaceae*. Interestingly, in our research, among the top 40 bacterial taxa identified by the SHAP algorithm, there was a species belonging to the *Erysipelatoclostridiaceae* taxa (*Erysipelotrichaceae UCG‐003*).^46^ Differential upregulated serum metabolites detected in this study were enriched around fatty acid biosynthesis (hit by 3‐oxododecanoic acid), and the association between fatty acid biosynthesis and diabetes, including T2D,^47^ as well as diabetic cardiomyopathy,^48^ has been investigated in previous studies.

In terms of differential lipid metabolites, the majority of phosphatidylcholines and lysophosphatidylcholines exhibited elevated levels in the GC group. Dysregulation of lipid and amino acid metabolism has been noted prior to the onset of islet autoimmunity in children who later develop T1D.^49^ Additionally, reduced levels of the PC class have been observed in early prediabetic NOD mice.^50^

Subsequently, we performed correlation analysis between these differentials and distinct microbial communities. In the correlation analysis, we observed a positive correlation between *Bacteroides_nordii*, *Bacteroides_cellulosilyticus*, and the 3‐oxolauric acid. Previous studies have indicated that lauric acid can exert antidiabetic effects,^51^ alleviate insulin resistance,^52^ and reduce hyperglycemia by restoring insulin and glucose homeostasis.^53^ This could be one of the indirect mechanisms through which the gut microbiota influences glycemic control. The upregulated lipid PC(38:2)(rep) in the GC group exhibited a negative correlation with *Bacteroides_coprocola*, which showed high abundance in the PGC group. This suggests its potential as a promising biomarker for predicting poor blood glucose control in patients with T1D.

In terms of clinical significance, the identification of specific gut microbes associated with glycemic control highlights the potential for microbiome‐based therapies in managing T1D. The positive correlations observed between specific microbial species and metabolites could lead to the development of personalized treatment strategies targeting gut microbiota to improve glycemic outcomes. Recent studies have increasingly focused on the role of gut microbiota in T1D, suggesting that alterations in the gut microbial community might contribute to the onset and progression of the disease. For instance, Murri et al. found that children with T1D had distinct gut microbial profiles compared to healthy controls,^54^ with lower diversity and different abundances of specific bacterial taxa. Similarly, Kostic et al. demonstrated that the gut microbiota undergoes dynamic changes preceding the onset of T1D,^55^ indicating a potential causal role. Despite these advances, the exact mechanisms by which gut microbiota influences T1D remain largely unexplored, particularly concerning how specific microbial species might affect glycemic control. Our findings suggest that the gut microbiota may serve as biomarkers for predicting glycemic control status, which could be valuable in the early identification of individuals at risk for poor glycemic control. Future research could aim to further elucidate the mechanisms through which these microbial species influence glycemic control and explore the therapeutic potential of modulating the gut microbiota in T1D. Additionally, large‐scale longitudinal studies are needed to validate our findings and assess the long‐term effects of microbiome‐metabolome‐lipidome‐targeted interventions in T1D management.

Our study has limitations. The cross‐sectional design prevented establishing causality, and despite a reasonable number of participants in our cohort, it was relatively small for this type of study. However, our cohort comprised carefully selected T1D patients with diverse disease durations and glycemic control, which allows our findings to be generalized to the majority of the diagnosed T1D population. In our study, the population exclusively consisted of T1D patients, which likely contributed to the observed similarity in microbial diversity between the GC and PGC groups. Given the T1D disease background, it is expected that microbial biodiversity does not show significant variation at a broad level. This consistency in biodiversity aligns with the homogeneity typically seen in cohorts with specific health conditions like T1D.^56^, ^57^ Moreover, the absence of significant differences in both α‐ diversity and β‐diversity analyses suggests that, although there may be compositional differences in specific microbial taxa, the overall gut microbiome diversity remains relatively stable between the two groups. This finding underscores the importance of focusing on the functional implications of specific microbial changes, rather than relying solely on general diversity metrics, to better understand their potential impact on glycemic control in T1D patients. In addition, dietary habits may differ between those with good glycemic control and those with poor glycemic control. Therefore, more studies are needed to determine if our results can be generalized to a larger population. A larger number of subjects would also serve to validate our findings. The untargeted profiling approach in our study unveiled numerous promising findings that enhance our comprehension of diabetic glycemic control pathophysiology. These warrant in‐depth exploration in larger prospective studies.

## 5 CONCLUSION

In conclusion, our study offers a comprehensive view of the relationship between gut microbiota composition, metabolomic characteristics, lipidomic profiles, and glucose control status in individuals with T1D. We have demonstrated that different levels of blood glucose control correlate with unique patterns in gut microbiota composition and circulating metabolites/lipids. Specifically, gut microbiota like *Bacteroides_cellulosilyticus* and *Bacteroides_coprocola*, associated with carbohydrate and fatty acid metabolism, show potential beneficial effects on varying blood glucose control levels in T1D, elucidated by microbiota‐related metabolites such as 3‐oxolauric acid. These insights deepen our understanding of the intricate interplay between blood glucose control, gut microbiota, and circulating metabolites/lipids, shedding light on their roles in T1D glycemic control. Furthermore, they pave the way for personalized targeted therapy strategies by modulating gut microbiota and associated microbial metabolites/lipids, potentially offering more effective and precise treatment avenues for T1D.

## AUTHOR CONTRIBUTIONS

This research was conceptualized and funded by JPW, XYZ, and SHL, who also provided supervision throughout the study. ZHG, LXP, HLT, TY, and YD directly accessed and validated the underlying data presented in the manuscript. Methodology and formal analysis were conducted by HLT, ZHG, LXP, TY, and YD. The manuscript was primarily drafted by HLT, ZHG, LXP, TY, and YD. All authors contributed to the article and approved the submitted version. SHL serves as the guarantor of this work, having full access to all study data and assuming responsibility for data integrity and accuracy of analysis. All authors critically reviewed the manuscript.

## FUNDING INFORMATION

The grants that supported this study are as follows: the Young Scientists Fund of the National Natural Science Foundation of China (82100822), the Strategic Priority Research Program of Chinese Academy of Sciences (Grant No. XDB38010100), the Program for Innovative Research Team of The First Affiliated Hospital of USTC (CXGG02), and the Anhui Provincial Natural Science Foundation (2008085MH248, 2008085MH278).

## CONFLICT OF INTEREST STATEMENT

The authors declare no conflicts of interest.

## CONSENT FOR PUBLICATION

All subjects provided written informed consent.

## Supporting information


**Data S1.** Supporting information.

## Data Availability

The data presented in this study are available on a reasonable request from the corresponding author. The datasets utilized in this research are available in public online databases. The specific repository and corresponding accession number are as follows: National Center for Biotechnology Information (NCBI) at https://www.ncbi.nlm.nih.gov/, with the accession number PRJNA766410.
