# Supplementary material for: Gut microbiota, serum metabolites, and lipids related to blood glucose control and type 1 diabetes
Source: J Diabetes. 2024 Oct 27;16(10):e70021. doi: 10.1111/1753-0407.70021 (PMC11513438; doi:10.1111/1753-0407.70021)
Supplement: Supplementary file 1 — Data S1. Supporting information. [file JDB-16-e70021-s001.pdf]

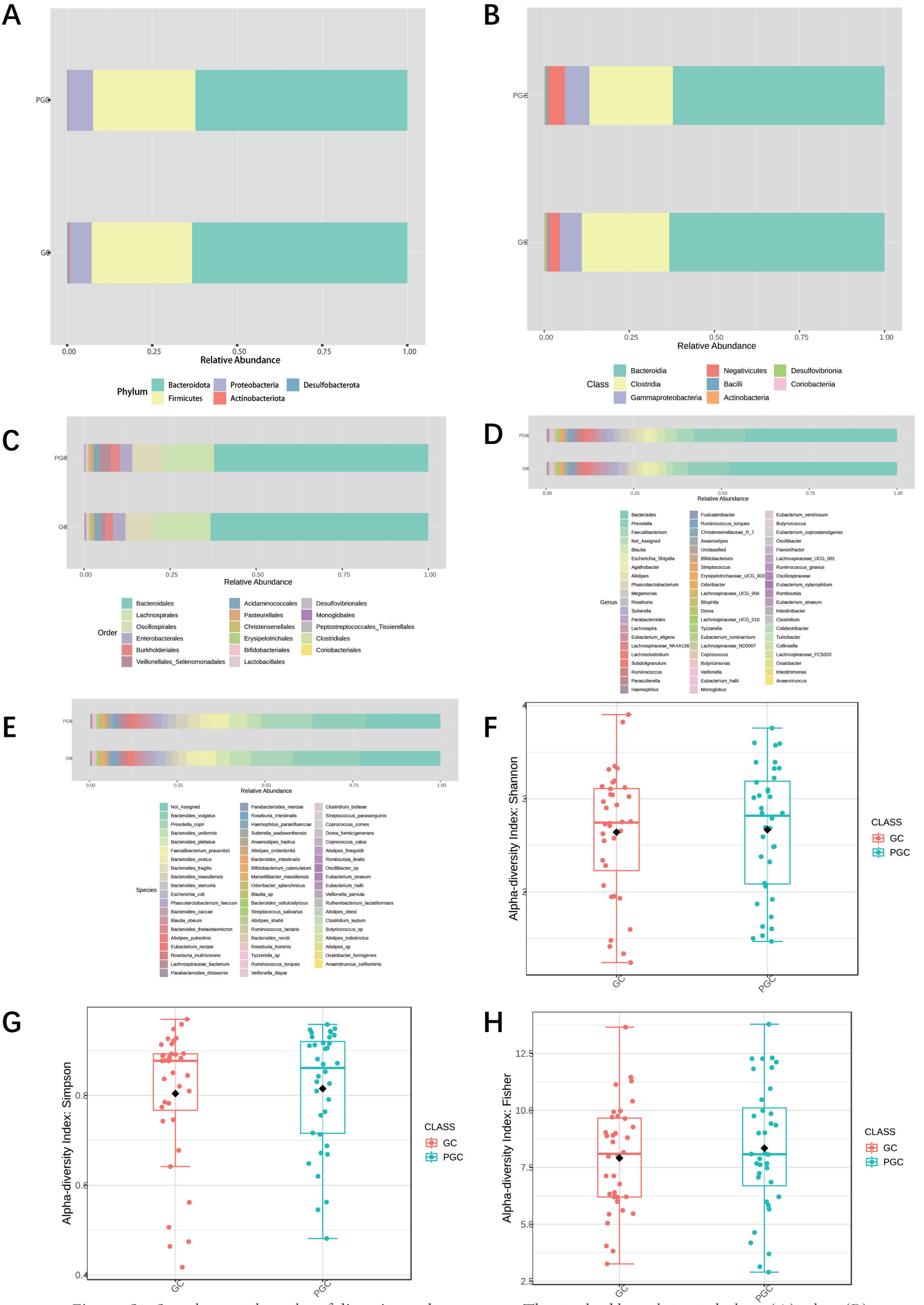

Figure. S1. Supplemental results of diversity and taxonomy. The stacked bar plots at phylum (A), class (B), order (C), genus (D), species (E) level. (F-H) The boxplots of α -diversity of Shannon(F), Simpson(G) and Fisher(H) index.

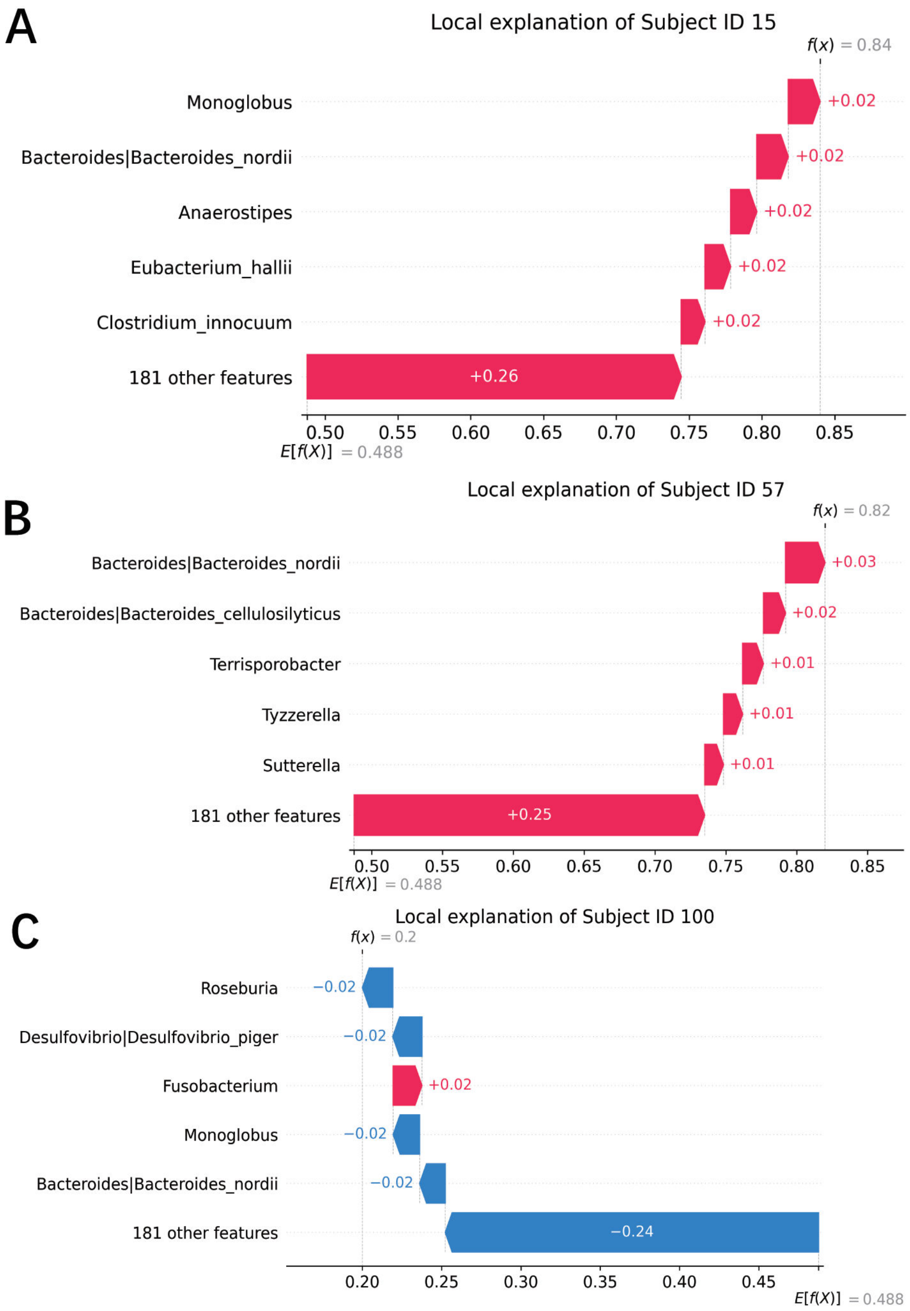

Figure. S2. The local explanations of three randomly chosen subjects of ID 15(A),ID 57(B) and ID 100(C) in GC and PGC groups.

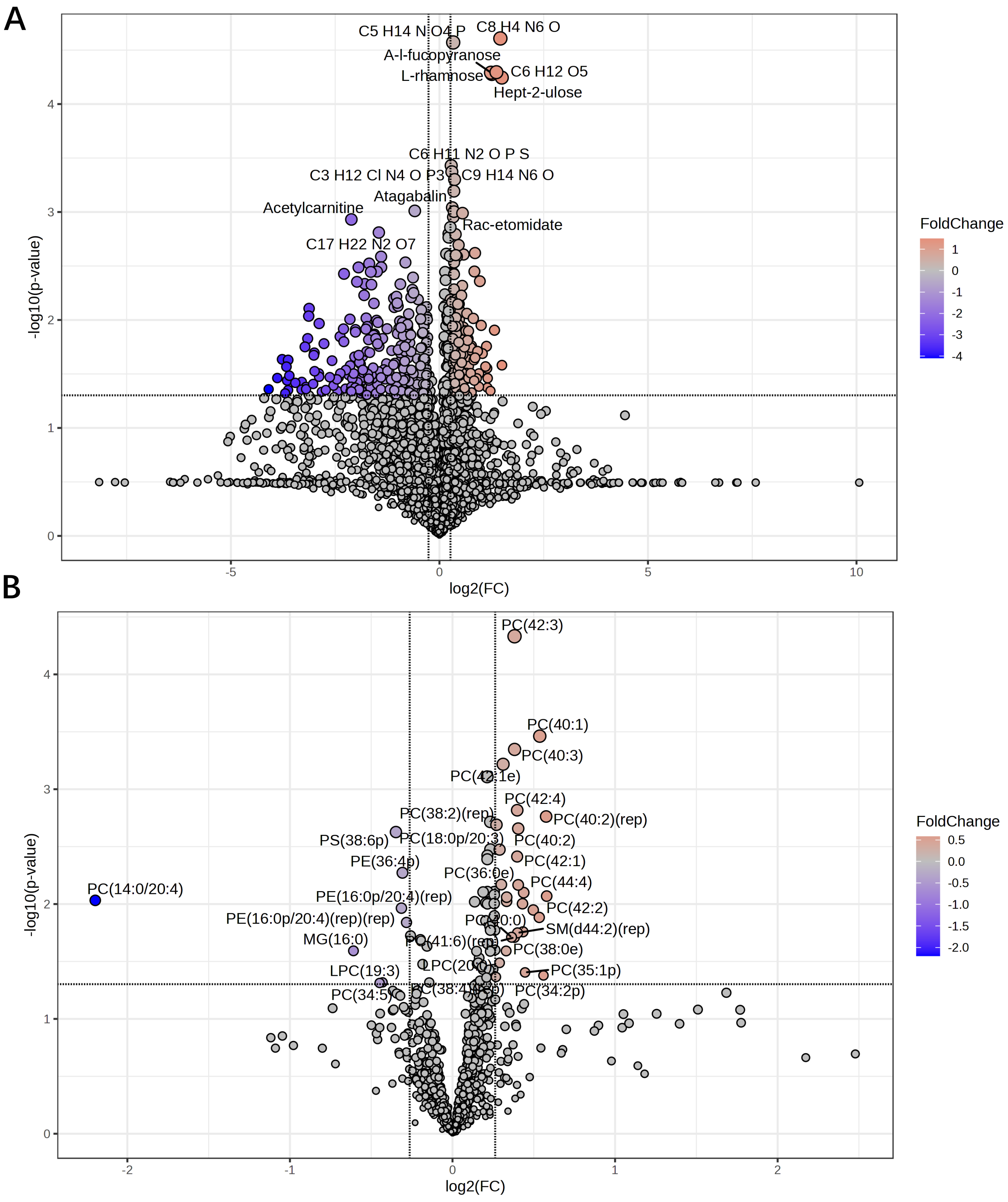

Figure.S3.Volcano plots showing the differential metabolites (A) and lipids (B) screened by combining P value (<0.05) andf old-change (>1.2). The significance was determined by using t-tests.

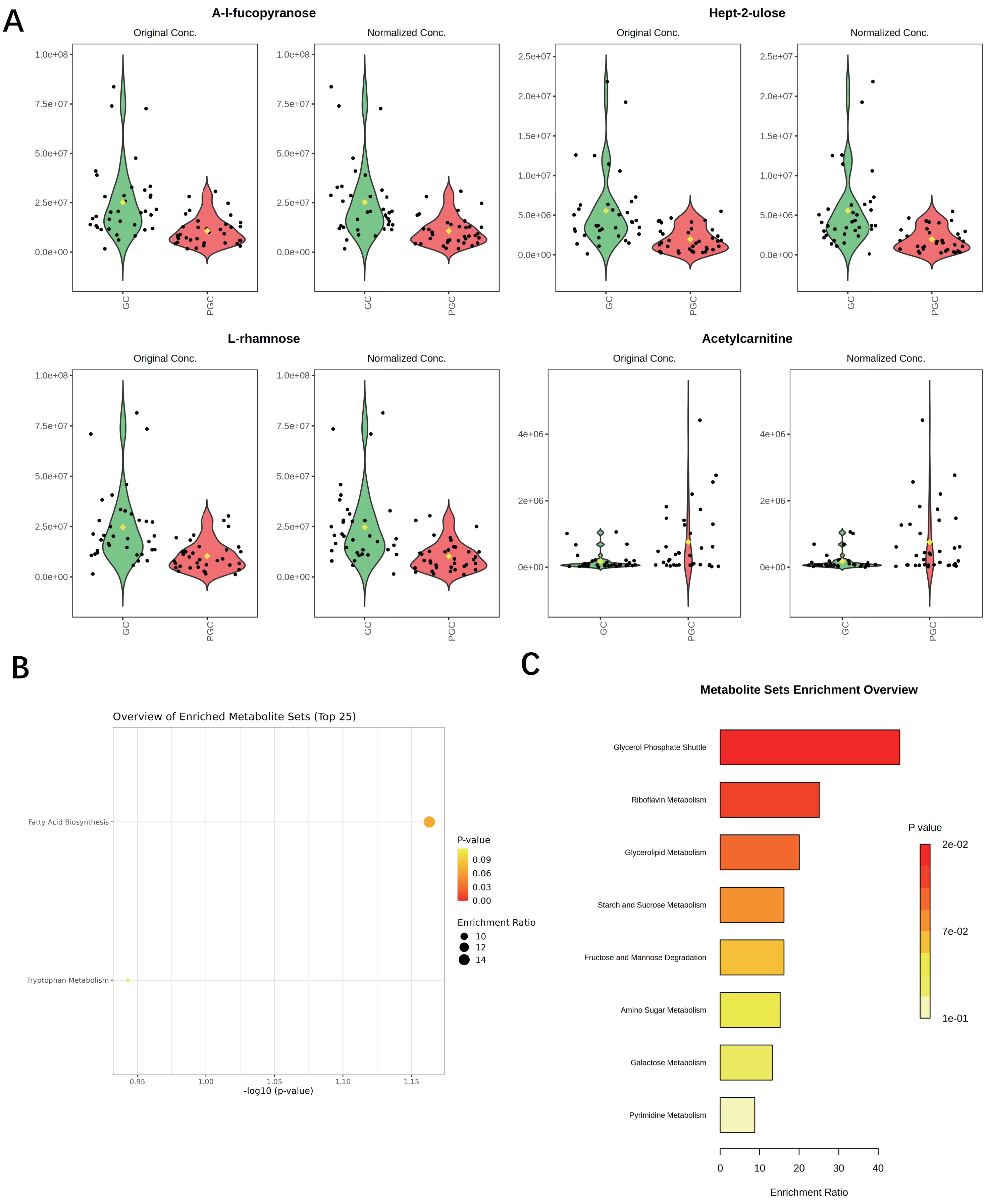

Figure.S4.The violin plots illustrating the differences between two groups for A-l-fucopyranose, Hept-2-ulose, L-rhamnose, and Acetylcarnitine. the violin plots (A).Enrichment sets of upregulated metabolites(B) and enrichment sets of downregulated metabolites(C).
